# Sensory and Cognitive Effects of Acute Exposure to Hydrogen Sulfide

**DOI:** 10.1289/ehp.10531

**Published:** 2007-10-30

**Authors:** Nancy Fiedler, Howard Kipen, Pamela Ohman-Strickland, Junfeng Zhang, Clifford Weisel, Robert Laumbach, Kathie Kelly-McNeil, Kelechi Olejeme, Paul Lioy

**Affiliations:** 1 Department of Environmental and Occupational Medicine and; 2 School of Public Health, University of Medicine and Dentistry of New Jersey–Robert Wood Johnson Medical School, Piscataway, New Jersey, USA; 3 School of Medicine, Morehouse University, Atlanta, Georgia, USA

**Keywords:** acute exposure, cognitive, hydrogen sulfide, sensory, symptoms

## Abstract

**Background:**

Some epidemiologic studies have reported compromised cognitive and sensory performance among individuals exposed to low concentrations of hydrogen sulfide (H_2_S).

**Objectives:**

We hypothesized a dose–response increase in symptom severity and reduction in sensory and cognitive performance in response to controlled H_2_S exposures.

**Methods:**

In separate exposure sessions administered in random order over three consecutive weeks, 74 healthy subjects [35 females, 39 males; mean age (± SD) = 24.7 ± 4.2; mean years of education = 16.5 ± 2.4], were exposed to 0.05, 0.5, and 5 ppm H_2_S. During each exposure session, subjects completed ratings and tests before H_2_S exposure (baseline) and during the final hour of the 2-hr exposure period.

**Results:**

Dose–response reduction in air quality and increases in ratings of odor intensity, irritation, and unpleasantness were observed. Total symptom severity was not significantly elevated across any exposure condition, but anxiety symptoms were significantly greater in the 5-ppm than in the 0.05-ppm condition. No dose–response effect was observed for sensory or cognitive measures. Verbal learning was compromised during each exposure condition.

**Conclusions:**

Although some symptoms increased with exposure, the magnitude of these changes was relatively minor. Increased anxiety was significantly related to ratings of irritation due to odor. Whether the effect on verbal learning represents a threshold effect of H_2_S or an effect due to fatigue across exposure requires further investigation. These acute effects in a healthy sample cannot be directly generalized to communities where individuals have other health conditions and concomitant exposures.

Workers and community residents employed by or living near industries such as pulp/paper, petroleum, and animal processing are exposed to hydrogen sulfide (H_2_S). At persistent high concentrations (> 500 ppm), H_2_S is lethal (e.g., Hendrickson et al. 2004). Furthermore, several studies suggest that chronic neurologic sequelae can occur from loss of consciousness due to transient, high-level H_2_S exposures (Deng and Chang 1987; Kilburn 1993; Parra et al. 1991; Snyder et al. 1995; Tvedt et al. 1991; Wang and Yu 1989). The neurologic effects of the lower H_2_S concentrations observed in communities, however, are less well understood and are in need of further investigation [Agency for Toxic Substances and Disease Registry (ATSDR) 2006; Woodall et al. 2005]. Therefore, the purpose of the present acute exposure study was to evaluate symptoms, odor and environmental quality ratings, and sensory and cognitive performance in response to three separate controlled exposures of 0.05, 0.5, and 5 ppm of H_2_S.

H_2_S metabolism occurs through oxidation, methylation, and reaction with metallo-or disulfide-containing proteins (Beauchamp et al. 1984; U.S. Environmental Protection Agency 1987). Animal studies suggest that H_2_S is rapidly and widely distributed but is not stored because of rapid metabolism and excretion (ATSDR 2006). Several underlying mechanisms that are as yet not well understood are likely responsible for the toxicity of H_2_S. For example, H_2_S has been shown to inhibit cytochrome oxidase in many tissues (Dorman et al. 2002). Moreover, Bhambhani and colleagues, in a series of human oral exposures to 5 and 10 ppm H_2_S, found decreased oxygen uptake, increased blood lactate, and decreased muscle citrate synthase activity. These effects were hypothesized as attributable to cytochrome oxidase inhibition (Bhambhani et al. 1996a, 1996b, 1997). However, toxicity due to cellular asphyxiation has been challenged by an animal model of H_2_S-induced apnea. From the results of this work, Almeida and Guidotti (1999) suggested that the apnea after a “knockdown” occurred as a result of an afferent neural signal from the lung via the vagus rather than a direct effect on the brain stem. Furthermore, Milby and Baselt (1999) observed that persistent neurologic effects among those experiencing acute H_2_S intoxication resulted from hypoxia secondary to respiratory insufficiency rather than a direct toxic effect on the brain.

Numerous epidemiologic studies (Beauchamp et al. 1984; Glass 1990; Haahtela et al. 1992; Jaakkola et al. 1990; Kangas et al. 1984; Mostaghni et al. 2000) document increased neurologic and respiratory symptoms among workers and community members exposed to intermittent and variable environmental and occupational concentrations of H_2_S. For example, relative to control communities, Legator et al. (2001) reported higher odds ratios for 9 of 12 symptom categories with the highest odds ratios for central nervous, respiratory, and blood systems (e.g., clotting disorder, bruising, anemia) among residents living in communities in Texas and Hawaii, where maximum 24-hr H_2_S concentration of 0.10–0.20 ppm and 0.20–0.50 ppm, respectively, were documented. Furthermore, a follow-up study of residents from Rotorua, New Zealand, where H_2_S exposure associated with geothermal energy was documented in the community (Bates et al. 1997), reported increased incidence of nervous system and sense organ diseases (Bates et al. 2002). However, exposure in these studies was often poorly characterized, involved mixtures of pollutants, and was highly variable among individuals.

Despite epidemiologic observations of neurologic symptoms associated with community exposure to H_2_S, relatively few objective studies of cognitive performance have been conducted. An exception is those studies performed by Kilburn and colleagues in which both sensory and cognitive performance were compromised among residents and workers chronically exposed to H_2_S (Kilburn 1997, 1999; Kilburn and Warshaw 1995). ATSDR also evaluated neurobehavioral performance among chronically exposed target residents who were estimated to have ≥ 0.09 ppm H_2_S exposure based on air monitoring data and modeling of exposure relative to a comparison group of residents whose exposure estimates were < 0.05 ppm. Although target residents exhibited marginally poorer performance on a test of memory, their overall neurobehavioral performance was similar to the comparison group (Inserra et al. 2004). Again, exposure estimates in these neurobehavioral studies were relatively imprecise and highly variable among subjects. Roth (1999) relies on the studies by Kilburn and Warshaw (1995) to suggest that chronic, low-dose exposure may produce persistent neurologic or cognitive effects. Based on the existing literature, we hypothesized significantly reduced environmental quality ratings, increased ratings of odor and symptom severity, and compromised sensory and cognitive performance according to a dose–response function.

## Methods

### Subjects

Seventy-four healthy nonsmokers (35 female; 39 male), with a mean (± SD) age of 24.7 ± 4.2 years and mean education of 16.5 ± 2.4 years were recruited from the UMDNJ–RWJ and Rutgers University community and surrounding New Jersey suburbs through advertisements in newspapers. Based on self-report, 47% (*n* = 35) were Asian, 35% (*n* = 26) Caucasian, 8% (*n* = 6) Hispanic, 7% (*n* = 5) African American, and 3% (*n* = 2) other. Subjects’ mean height was 66.3 ± 3.5 inches, mean weight was 154.2 ± 33.1 lb, and mean body surface area was 1.8 ± 0.2 (Mosteller 1987). When subjects contacted the research office, they were given a brief explanation of the study, and if they remained interested in participation, verbal consent for telephone screening was obtained. Eighty-one percent of those who called about the study passed the initial screening (156 of 193). Of the 156 who passed the initial screening, 47% (74) completed the protocol, 2% (3) dropped out before completion, 13% (21) did not pass the physical examination, 9% (14) had scheduling conflicts and could not participate, 24% (37) changed their mind, and 5% (7) were lost to follow-up. Subjects were excluded from participation during the screening or medical examination for the following conditions: neurologic disease or brain injury, significant ongoing or previous exposure to other neurotoxicants such as lead and pesticides, stroke or cardiovascular disease, serious pulmonary disease (e.g., asthma), liver or kidney disease, serious gastrointestinal disorders (e.g., colitis), major psychiatric conditions including psychoses, bipolar disorder, alcoholism, or drug abuse, or use of certain medications (e.g., anxiolytics, antidepressants, beta blockers). No pregnant or lactating women were included in the study. Subjects who met the initial screening criteria were scheduled for a physical examination. All recruitment and testing procedures were reviewed and approved by the Institutional Review Board of the University of Medicine and Dentistry of New Jersey. Subjects were paid a total of $450 for completion of the study.

### Screening examination

**Figure 1:**
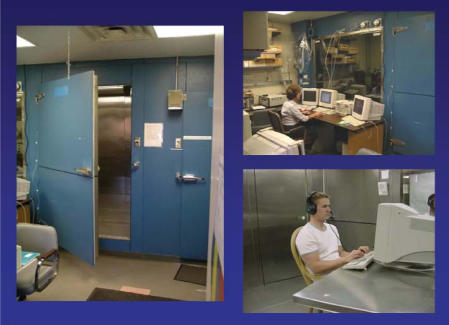
Controlled environment facility: technician and exposure stations.

Before participation, subjects were sent information about the study requirements and the informed consent document. On arrival, subjects were taken to a room where they were given a verbal explanation of the study and the opportunity to ask and have questions answered before signing the consent. Subjects then completed a medical history and physical examination by an occupational physician and performed spirometry, electrocardiogram (EKG), blood counts and routine chemistries, and visual acuity testing. To reduce anxiety, subjects were shown the controlled environment facility (CEF), where they were taught the procedures for the exposure conditions (Figure 1).

### Inhalation exposure

Administering three concentrations of H_2_S determined the dose–response function with exposure concentrations that *a*) fell below the estimated, minimal risk level (< 0.07 ppm) (ATSDR 1999); *b*) encompassed the environmental concentrations associated with symptoms (0.5 ppm); and *c*) included the recommended American Conference of Governmental Industrial Hygienists occupational threshold limit value of 5 ppm. The latter is the allowable exposure concentration for workers expected to be exposed for 8 hr/day, 5 days/week, over a 40-year working lifetime.

For H_2_S delivery and analysis, all gases were certified on grade or concentrations. All tubing and connections were stainless steel or Teflon. H_2_S gas was delivered to the CEF at 99.3% pure gas for environmental conditions of 5 ppm. For the lower environmental concentrations (i.e., 0.05 and 0.5 ppm), a 1% (10,000 ppm) H_2_S mix in air was delivered. The H_2_S gas flow rate into the system was controlled using a Cole-Parmer Precision Gas Flow Meter (model no. 32915–88; Cole-Parmer, Vernon Hills, IL). The total flow through the CEF was 300 ft^3^/min. Concentrations were monitored via a Teflon sampling line attached to the sample inlet port on the rear of the H_2_S analyzer. The CEF operated at slightly negative pressure to minimize any possibility of H_2_S being distributed into the control room and waiting area. The H_2_S concentration within the CEF followed an exponential increase, reaching its targeted concentration within 10 min. The concentration was maintained within ± 10% of the target value by a computer-controlled system during that time period and was measured continuously using an API Model 101A H_2_S analyzer (Advanced Pollution Instrumentation, Inc., San Diego, CA). The instrument was calibrated monthly with a 10-ppm H_2_S cylinder and a zero-grade air cylinder.

### Behavioral measures

#### Odor ratings, symptoms, and environmental quality (Table 1)

Subjects completed analog scales to rate pleasantness, intensity, and irritation of the H_2_S odor, and to evaluate environmental qualities. Symptoms were rated on a ratio scale from 0 (barely detectable/no sensation) to 100 (strongest imaginable) (Green et al. 1996). The symptoms chosen were based on those used in community and environmental studies of H_2_S (Jaakkola et al. 1990; Legator et al. 2001).

#### Sensory Function

Postural sway has been sensitive to acute and chronic effects of several neurotoxicants (Bhattacharya et al. 1987; Dick et al. 1999, 2001; Sack et al. 1993) including H_2_S (Kilburn and Warshaw 1995). We used protocols developed by the National Institute for Occupational Safety and Health (Dick et al. 1990, 1999; National Institute for Occupational Safety and Health 1995) and the University of Cincinnati (Bhattacharya et al. 1987, 1995), to assess postural sway. A computerized biomechanics platform system [AccuSway Computerized Platform System; Advanced Mechanical Technology, Inc. (AMTI), Watertown, MA] consisted of a 50 × 50 cm platform with temperature-compensating strain gauge transducers and a signal conditioner/amplifier. Subjects were instructed to stand on the platform (without shoes), arms at their sides, heels together, with feet at a 30° separation angle, which was maintained by footprints marked on the platform surface. Test procedures assessed the effect of vision, proprioception, and the vestibular system on postural stability. Six test conditions repeated twice, each lasting 30 sec and preceded by one practice trial, were used. In four conditions, subjects stood on two legs, eyes open and closed, with and without a 4-inch foam pad; and in two conditions they stood on one leg (right and then left leg, independently). AMTI software was used for data collection and calculation of sway area (square centimeters) and sway length (centimeters).

#### Visual acuity and visual contrast sensitivity (VCS)

Loss of sensitivity to visual contrast in the intermediate range has been observed among workers with chronic exposure to solvents (Donoghue et al. 1995; Frenette et al. 1991; Hudnell et al. 1996a, 1996b; Mergler et al. 1991). Jarvinen and Hyvarinen (1997) also reported acute loss of contrast sensitivity during the workday among workers exposed to triethylamine. This study suggests that ocular sensory irritation, such as that documented with H_2_S, may result in decrements in visual contrast sensitivity. Subjects must have adequately corrected visual acuity (20/40 or better) and no major illness (e.g., diabetes) to obtain valid results for contrast sensitivity. For the contrast sensitivity test, presentation of the five grating frequencies (1.5, row A; 3, row B; 6, row C; 12, row D; 18, row E ) were randomized and presented starting with the right and then the left eye. The entire sequence was presented twice for each eye. Within each row there are nine patches. Each patch is converted to a contrast value assigned by the test manufacturer. The final contrast sensitivity score for each frequency or row is determined by the mean of the lowest contrast patch having at least two of three correct responses.

### Cognitive tests

To reduce the effects of practice from repeated administration of the neurobehavioral tests, alternate forms were developed. That is, different yet equivalent stimulus materials were used during each of the six test administrations (e.g., six alternate word lists). These materials were obtained from the test authors and were piloted according to our within-subject exposure design before onset of the actual exposure study. In addition, subjects were trained in the test procedures before exposure sessions to minimize practice effects.

#### Simple Reaction Time (SRT) and Continuous Performance Test (CPT)

Simple and complex reaction time tests (Letz 1998) are among the visuomotor tests most sensitive to subtle effects of neurotoxicants (Dick 1995; Gamberale 1985) including H_2_S (Kilburn 1999; Kilburn and Warshaw 1995). To assess simple visual reaction time, we instructed subjects to press a button on the keyboard as quickly as possible whenever a stimulus in the form of a large square appeared on the computer screen. The amount of time between stimuli varied, thus minimizing the possibility of stimulus anticipation. Subjects performed the test once with the dominant hand, followed by a trial with the nondominant hand. Reaction time latencies were measured and recorded by the program. For the CPT, letters flashed at a rate of one per second on the computer screen. Subjects were instructed to press the response button when the target letter flashed on the screen. Eighty target stimuli were embedded within eight blocks of letters (10 stimuli/block). The letter chosen as the target stimulus and the location of the target stimuli within a block of stimuli were varied to develop alternate forms.

#### Finger Tapping test

Motor speed is one aspect of performance that may be slowed by exposure to neurotoxicants such as alcohol (Savolainen 1980). On this test of motor speed (Letz 1998), the subject was instructed to tap a keyboard button as many times as possible within a 30-sec interval. The subject was administered a total of four trials: two with the dominant hand, one with the nondominant hand, and one with both hands. When both hands were used, the subject was required to alternately tap two buttons on the keyboard. The subject must tap a minimum of 25 taps within each trial. The number of button presses was recorded separately for each trial.

#### Symbol-Digit Substitution test (SDS)

This test is sensitive to the effects of H_2_S (Kilburn and Warshaw 1995) and has been moderately sensitive to the acute effects of laboratory exposures to neurotoxicants (Dick 1995). The SDS (Letz 1998) is a test of perceptual–motor functioning, requiring motor persistence, sustained attention, response speed, and visuo-motor coordination. For this test, a “key” was presented at the top of the computer screen which consists of nine digits and nine corresponding symbols. The subject was instructed to press the digits on the keyboard that corresponded with a test set of nine symbols presented in scrambled order. A total of seven sets of digit-symbol pairs were presented to the subject. The response latency for each of the nine items in each trial was recorded, as well as the number of incorrectly matched digits and symbols.

#### Auditory Verbal Learning Test (AVLT)

Kilburn and colleagues (1995) reported verbal recall as compromised by exposure to H_2_S at low concentrations (0.1 to 1ppm). On the AVLT (Crawford et al. 1989), a list of 15 words (List A) was presented verbally by the examiner through headphones (one word is presented per 3 sec) five consecutive times (Trials 1–5). After each trial, subjects were instructed to verbally recall as many words as possible. An interference list of 15 items was then presented (Trial 6) (List B), and subjects were requested to recall these words. Thirty minutes later, subjects were administered a sixth recall trial of the first list, as well as a 50-word recognition list. The test has six alternate forms; two of the alternate word lists have retest reliability between 0.60–0.77 (Ryan et al. 1986). Furthermore, no significant performance improvements occurred when subjects were retested with different forms (Crawford et al. 1989).

### Knowledge of exposure

After each exposure, subjects were asked to “guess” whether their exposure to H_2_S was at the lowest, medium, or highest concentration (Hughes and Krahn 1985).

## Procedure

**Figure 2:**
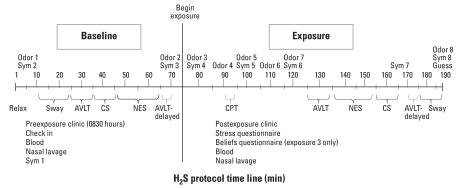
Timeline of events during each exposure session. Abbreviations: CS, contrast sensitivity vision test; Guess, exposure guess questionnaire; NES, Continuous Performance, Simple Reaction Time, Finger Tapping, Symbol Digit Substitution; Sym, symptom.

Each experimental session was 3 hr in duration and occurred in the morning to control for the effects of circadian rhythms (Figure 2). On the day before each testing session and on the day of testing, subjects were asked not to use caffeine or alcohol. Subjects also could not have an active upper respiratory illness (either infection or allergy) nor use medication for allergies or other respiratory conditions for 1 week before the onset of the study or during the study. For subject safety, EKG electrodes monitored heart rate and variability during all exposure conditions. On the day of each experimental session, subjects reported to the Clinical Center at 0830, and a nurse performed a check-in to ascertain that the above conditions were met. Women were given a pregnancy test. Subjects completed the symptom questionnaire (clinic baseline) and then were escorted to the CEF where they were seated in a standard nonpadded office chair. Subjects rested quietly for 5 min, after which they completed the symptom questionnaire and odor and environmental ratings (10 min), and performed the sway test, contrast sensitivity test, and the cognitive tasks (baseline). (All times given are the approximate times after each task began.) Neither subjects nor the experimenters responsible for monitoring subjects and conducting each session were told the exposure conditions, and subjects were randomly assigned to one of six possible exposure orders (e.g., 5, 0.05, 0.5 ppm). However, the exposure technician was aware of the exposure condition in order to monitor exposure concentrations on each day. On completion of all tasks, the symptom questionnaire and odor and environmental ratings (70 min) were administered before exposure began. After the onset of exposure when exposure concentrations reached a steady state, the symptom questionnaire and odor and environmental ratings (80 min) were completed to obtain an immediate response to the odor of the exposure. During the next 10 min, subjects were asked to relax and read magazines provided and then to complete the odor ratings (90 min). During the next 5-min period, subjects completed the CPT task. The symptom questionnaire and odor and environmental ratings (100 min) were then completed and subjects were allowed to read for 10 min. After completion of the odor and environmental ratings (110 min), the subject read for 10 min and then completed odor and environmental ratings and symptom questionnaire (120 min). After 45 minutes of exposure, the sway test, contrast sensitivity, and cognitive tests required approximately 60 min to complete while exposure was ongoing. Subjects also completed another symptom questionnaire (165 min). Finally, subjects completed the symptom questionnaire and odor and environmental ratings (180 min) and were asked to guess the exposure condition. Immediately on termination of the protocol (~ 3 hr), subjects returned to the Clinical Center, removed the electrodes for heart monitoring, and completed the symptom questionnaire (clinic recovery). Subjects were allowed to leave the Clinical Center if their symptoms returned to the same level as recorded at baseline before exposure.

## Statistical Analysis

### Odor ratings, symptoms, and environmental qualities

Ratings of odor, symptoms, and environmental qualities at 70 min (before exposure onset) served as the baseline. All subsequent odor ratings, symptoms, and environmental qualities were compared with their respective baselines. We created a total symptom severity score for each time point by adding ratings for all symptoms. We created scores for subscales of symptoms by adding symptom severities for each symptom within the subscales (see Table 1). Ratings of odor intensity, irritation, and pleasantness were each analyzed separately, as were ratings for each environmental quality (Table 1). We used mixed linear models to test the effect of exposure × time, and type 3 *F*-tests to test the significance of the interaction (Liang and Zeger 1986). Time was entered into the model as a categorical variable. Contrasts were used to test whether individual changes in ratings from baseline to each subsequent time point differed between the three exposure conditions. This analysis was first completed for the total mean symptom severity and then for each subscale of symptoms. We report uncorrected alpha values, with the alpha level after Bonferroni correction noted for each group of multiple comparisons.

### Sensory and neurobehavioral tests

We used descriptive statistics to examine the distribution of the variables for each neurobehavioral test. Data for all neurobehavioral measures were normally distributed; therefore, we used linear mixed models for analysis. Type 3 *F*-tests were used to test significance of the interaction between exposure and time, representing the effect of exposure on a change in response from baseline to during exposure.

Contrast sensitivity values for the five grating frequencies (rows) were highly concentrated on one value per row with dispersion across other values. Therefore, original data were transformed to binomial values of 0 (not perfect) or 1 (perfect) for each row separately. We used generalized linear mixed models with logit links and type 3 *F*-tests to test the significance of the interaction between exposure and time. A random effect accounted for similarities between responses from the same individual during different sessions, whereas a repeated measures correlation structure was used to account for the correlation between responses from the same individual within a session. We conducted the estimation and hypothesis testing using the SAS (version 9.1.3; SAS Institute Inc., Cary, NC) programming language (Proc mixed for continuous responses and Proc GLM mixed for binary responses).

## Results

### Odor ratings, symptoms, and environmental quality ratings

After controlling for baseline, we observed a significant exposure × time interaction for odor ratings of irritation (*F* = 4.92; df = 12, 1088; *p* < 0.0001), intensity (*F* = 24.58; df = 12, 1093; *p* < 0.0001), and pleasantness (*F* = 9.86; df = 12, 1093; *p* < 0.0001). The first degree of freedom is based on the number of exposures (3 – 1) times the number of time points (7 – 1); the second degree of freedom is the number of observations minus the number of fixed effect parameters minus the random effects parameters. (Differing degrees of freedom are a result of missing data points for certain ratings.) With Bonferroni correction, analyses controlling for baseline and comparing each time point between exposure conditions revealed significantly increased ratings of intensity at 80 (exposure onset) and 90 min for pairwise comparison of all exposure conditions and for pairwise comparisons at 100 min of 0.05 ppm to 0.5 ppm and of 0.05 ppm to 5 ppm. At 110 min, ratings of intensity were significantly increased at 5 ppm relative to 0.05 ppm. With the same analytic strategy, ratings of irritation and pleasantness at 80 min (exposure onset) were significantly greater when comparing 0.05 ppm to 0.5 ppm and 0.05 ppm to 5 ppm. Ratings of irritation were also significantly greater when comparing ratings at 90 min during the 5-ppm relative to the 0.05-ppm conditions (data not shown). Ratings of odor were elevated immediately after exposure onset and then began to wane consistent with habituation to the odor.

**Figure 3:**
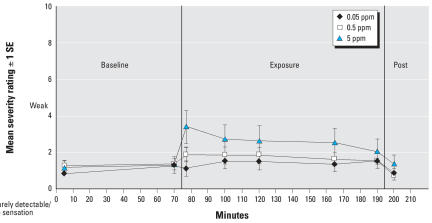
Mean anxiety symptom severity at each time point across exposure conditions. Post, postexposure. Change from 70 min to 80 min; 0.05 vs. 5 ppm: *p* = < 0.0001. Change from 70 min to 100 min; 0.05 vs. 5 ppm: *p* = 0.01. Change from 70 min to 120 min: 0.05 vs. 5 ppm; *p* = 0.03. Change from 70 min to 165 min: 0.05 vs. 5 ppm: *p* = 0.02; 0.5 vs. 5 ppm: *p* = 0.04. Error bars represent SE.

We observed a significant exposure × time interaction for the anxiety subscale (*F* = 2.52; df = 12, 1087; *p* = 0.003) (Figure 3) but not for total symptom severity or any other symptom subscale. Following the same analytic strategy, we performed pairwise comparisons of exposure conditions for total symptom severity and all subscales after controlling for baseline symptom scores in each exposure condition. The only significant difference observed with Bonferroni correction revealed significantly greater anxiety at 80 min in the 5-ppm relative to the 0.05-ppm condition (*p* < 0.0001). Without Bonferroni correction (i.e., *p* < 0.05), subjects reported significantly greater total symptom severity at 80, 100, 120, and 165 min when comparing the 0.05-ppm to the 5-ppm exposure conditions (not shown). For those time points when the total severity score was significantly different, subscales were also compared, again controlling for baseline scores. At 80 min, subjects reported significantly more anxiety, lower respiratory symptoms, and cognitive symptoms in the 5-ppm relative to the 0.05-ppm condition. Symptoms of anxiety, lower respiratory, and cognitive were significantly greater at 100 min in the 5-ppm relative to the 0.05-ppm condition. At 120 min, subjects reported significantly more general somatic symptoms, anxiety, and lower and upper respiratory symptoms in the 5-ppm relative to the 0.05-ppm condition. Upper respiratory symptoms were also significantly greater at 165 min in the 5-ppm relative to the 0.5-ppm condition. Anxiety continued to persist at 165 min, with greater anxiety reported in the 5-ppm relative to the 0.05-ppm and the 0.5-ppm conditions. However, symptoms measured after the neurobehavioral testing (190 min) were not significantly elevated for any comparison.

The overall test of the exposure × time interaction revealed a significant effect for ratings of air quality (*F* = 7.84; df = 12, 1083; *p* < 0.0001), odor quality (*F* = 11.89; df = 12, 1085; *p* < 0.0001), and the need to ventilate the room (*F* = 8.20; df = 12, 1083; *p* < 0.0001). With Bonferroni correction of pairwise comparisons, subjects reported significantly poorer air quality, odor quality, and a greater need to ventilate the room at 80 min in the 0.5-ppm and 5-ppm conditions relative to the 0.05-ppm condition. Air and odor quality continued to be significantly more negatively evaluated at 100 min in the 5-ppm relative to the 0.05-ppm condition. Similar to odor ratings, negative ratings of environmental qualities were highest early during exposure and then began to wane as exposure continued (data not shown).

### Sensory and cognitive tests

#### Contrast sensitivity

With the general linear mixed model using binomial results for the contrast sensitivity at all spatial frequencies, the interaction of exposure × time was not significant for the right or left eye. Likewise, no significant main effects of exposure or of time for the right or left eye were observed (results not shown).

#### Sway

The mixed linear model revealed a significant exposure × time effect on sway length in the right leg only condition. Controlling for baseline, pairwise comparisons revealed a significantly decreased sway length during exposure to the 0.5-ppm condition relative to the 0.05-ppm condition and a trend toward decreased sway length in the 5-ppm condition relative to the 0.05-ppm condition. We also observed a trend toward significance of exposure × time for sway length, left leg, and for sway area, eyes open. The exposure main effect was significant for sway area, eyes closed and eyes open, and soft surface, and approached significance for sway length, eyes closed. Time had a significant effect during exposure relative to preexposure for sway length eyes open, soft surface, and for eyes closed, soft surface. For all of these sway parameters, performance was slightly improved during exposure, showing less area and length of sway (Tables 2 and 3).

#### Finger Tapping

The general linear mixed model did not reveal any significant exposure × time interaction for the number of finger taps with the preferred hand (*F* = 0.89; df = 2, 216; *p* = 0.41), nonpreferred hand (*F* = 0.42; df = 2, 216; *p* = 0.42), or alternating (*F* = 0.13; df = 2, 216; *p* = 0.14) hands. No main effect of exposure or time was identified for the nonpreferred or alternating hands (data not shown), but a significant effect of time was shown for the preferred hand, revealing a small increase or improvement in the number of taps from baseline to exposure (*F* = 6.10; df = 1, 216; *p* < 0.01).

#### Simple and complex reaction time

We observed no significant exposure × time interaction with the general linear mixed model for simple (*F* = 0.24; df = 2, 216; *p* = 0.79) or complex reaction time (*F* = 1.01; df = 4, 433; *p* = 0.40). We observed no main effect of exposure or time for simple reaction time and for exposure for complex reaction time (data not shown). However, a significant effect of time was observed for complex reaction time with an increase or slowing in latency of response during exposure relative to baseline (*F* = 12.92; df = 2, 433; *p* < 0.0001). Specifically, latency of response was increased at the 90-min measurement during exposure, with the only trend toward significance in the pairwise comparisons of the change in the 0.5-ppm to the 5-ppm condition (*F* = 3.55; df = 1, 433; *p* = 0.06) (data not shown).

#### SDS

No significant exposure × time interaction was revealed for latency of response in the symbol digit substitution task (*F* = 0.79; df = 2, 217; *p* = 0.46). The exposure main effect was also not significant, but there was a trend toward significance for the time effect with a small reduction or improvement in latency of response during exposure (*F* = 3.45; df = 1, 217; *p* = 0.06) (data not shown).

#### AVLT

**Figure 4:**
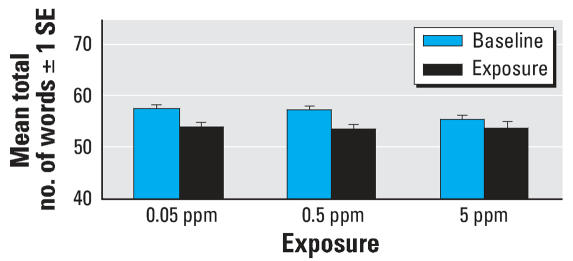
Mean total number of words recalled from List A over 5 trials on the AVLT. Change from baseline to during exposure: *p* < 0.0001.

The mixed linear model did not reveal any significant exposure × time interaction for recall of List A after presentation of the interfering List B, recall of List A after a 30-min delay, or recall of List B. A marginal trend was observed for total List A recall (*F* = 2.51; df = 2, 219; *p* = 0.08) with a somewhat larger effect of exposure in the 0.05- and 0.5-ppm conditions (Figure 4). Based on the results of the main effects analyses, it appears that although there was no main effect of exposure for any of the AVLT variables: total List A recall (*F* = 35.43; df = 1, 219; *p* < 0.0001), recall of List A after presentation of the interfering List B (*F* = 13.30; df = 1, 219; *p* = 0.0003), and recall of List A after a 30-min delay (*F* = 75.88; df = 1, 219; *p* < 0.0001) were all significantly worse during exposure, illustrating a significant time effect. This time effect appears to be more obvious for the 0.05-ppm and 0.5-ppm conditions relative to the 5-ppm condition. Furthermore, when comparing recall of Trial 1, List A, to recall of List B, no evidence of proactive interference was observed during exposure. That is, subjects either improved or performed similarly in their recall of List B relative to Trial 1 of List A.

#### Knowledge of exposure

Subjects were more likely to correctly identify the lowest and highest exposure concentrations accurately [0.05-ppm accuracy = 58% (*n* = 43); 0.5-ppm accuracy = 39% (*n* = 29); 5-ppm accuracy = 42% (*n* = 31); Cochran–Mantel-Haenszel, *p* < 0.0001)]. Furthermore, when the actual exposure condition was correlated with the “guess” made at that exposure within each exposure session (i.e., controlling for order of exposure), the percent agreement improved from 32.4% at exposure 1, to 48.6% at exposure 2, to 51.1% at exposure 3 (visit 1 kappa = –0.02; visit 2 kappa = 0.25; visit 3 kappa = 0.37).

## Discussion

H_2_S is well known for its low odor threshold and noxious valence for most humans. Other than the neurobehavioral studies of chronic exposure (Inserra et al. 2004; Kilburn 1997, 1999; Kilburn and Warshaw 1995), no other studies have evaluated the neurobehavioral effects of H_2_S in the absence of other pollutants and at concentrations documented to occur environmentally and occupationally. Under carefully controlled conditions, our study confirmed that even at environmental concentrations of H_2_S as low as 0.05 ppm, subjects rate the odor as more intense, irritating, and unpleasant than baseline room air, and they rate air quality as degraded. Furthermore, anxiety symptoms were significantly increased at exposure onset, with greatest absolute increases at onset of the 5-ppm exposure condition.

Counter to our hypothesis, no dose–response effect was observed for any of the sensory and cognitive measures of performance. However, performance on the verbal learning task declined from baseline in all exposure conditions, suggesting that subject fatigue or lapses in the ability to maintain attention to the material during the exposure period, rather than H_2_S exposure, could account for the finding. Separate post hoc covariance analyses controlling for self-reported fatigue, drowsiness, and concentration did not change the statistical significance for verbal learning (data not shown). The decline in verbal learning across all exposure conditions was intriguing because of its consistency with observational studies of chronic exposure. For example, Inserra et al. (2004) reported a marginal decline in performance on a delayed match to sample memory task, and Kilburn and colleagues (Kilburn 1999; Kilburn and Warshaw 1995) also reported reduced verbal memory performance among selected samples of workers and community members relative to an unexposed normative sample. Although our data are consistent with a threshold effect of H_2_S as low as 0.05 ppm, such an effect was not consistently observed for other neurobehavioral measures. For example, although latency of response was slowed for complex reaction time 15 min after exposure onset this effect was observed only in the 0.5-ppm condition relative to the 0.05-ppm condition. Furthermore, later during exposure (140 min), CPT performance was not significantly altered for any exposure condition. Second, symbol digit latency of response, a measure that relies on working memory, showed a small improvement with exposure, as did simple motor speed assessed with finger tapping, preferred hand. In contrast, Kilburn and colleagues (Kilburn 1999; Kilburn and Warshaw 1995) reported consistently compromised neurobehavioral performance across several domains of function. This issue is ultimately unresolved by the present study.

Odor ratings were significantly increased at onset of exposure, and the absolute value of these ratings increased with increasing concentration of H_2_S. Although these odor ratings moderated during exposure, they did not return to the baseline levels until after termination of exposure, suggesting that habituation may not be complete even at the lowest concentration of H_2_S (0.05 ppm). Consistent with odor ratings, ratings of environmental qualities show a similar pattern, with the greatest negative air and odor quality reported at the onset of exposure for the 5-ppm exposure condition. These findings suggest that subjects may not fully habituate to the smell of H_2_S at least during a 2-hr period, and therefore continuing exposure may prove to be annoying over time.

Subjects did not simply report more symptoms indiscriminately, as evidenced by the lack of overall increased total symptom severity. Although the onset of exposure induced the greatest increase in symptoms such as anxiety, poor concentration/confusion (cognitive), and shortness of breath/chest tightness (lower respiratory), the actual differences detected were on the order of a one- (lower respiratory) or two (anxiety)-point increase on a 100-point rating scale. These increases in symptom severity cannot be regarded as clinically significant. Furthermore, a post hoc analysis examining the effect of exposure on anxiety symptoms, in which concurrent ratings of odor irritation were covaried, revealed no significant exposure × time interaction effect on anxiety (*F* = 0.92; df = 8, 641; *p* = 0.5). Moreover, changes in the severity of irritation due to odor significantly affected changes in anxiety (*F* = 54.23; df = 1, 641; *p* < 0001). Thus, it appears that the odor of exposure had a significant influence on anxiety reported by subjects.

Like odor ratings, symptoms waned with time but did not return to baseline, although it is of interest that symptom reports were not exacerbated by performance of the neurobehavioral tests. In fact, symptom reports were no longer significantly different between exposure conditions immediately after neurobehavioral testing but before exposure ended. It also appears that a greater severity of physical symptoms was reported as exposure progressed (100 min) to include those associated with general physical discomfort (e.g., skin irritation, body aches), eye irritation, lower and upper respiratory discomfort (e.g., nasal congestion, throat irritation, shortness of breath/chest tightness), and headache/fatigue and nausea. These symptoms are also consistent with those reported in epidemiologic studies of H_2_S (e.g., Jaakola et al. 1990). Although several respiratory symptoms have been reported in epidemiologic studies as well as in our controlled exposure study, controlled exposure studies by Bhambhani and colleagues (1994, 1996a, 1996b) did not reveal significant differences in respiratory physiologic parameters (e.g., oxygen uptake) or in lung function among healthy male and female subjects exposed to 5 and 10 ppm H_2_S with and without exercise.

Inevitably, interpretation of our study is limited by the omission of a 0-ppm exposure condition and questions regarding the statistical power we had to detect significant effects. Ratings of odor, environmental qualities, and anxiety showed the greatest effect of exposure. Post hoc power analyses indicate that we had a 95.6% ability to detect a 10-point difference in lower respiratory symptoms from baseline relative to 80, 100, and 120 min for the 5-ppm exposure condition, and a 5-point increase in lower respiratory symptoms at those time points during the 0.5-ppm exposure condition. Thus, our study was adequately powered to find symptom severity of greater magnitude than was observed in the present study.

Our findings cannot be directly generalized to communities or workers chronically exposed to H_2_S alone or in combination with other pollutants. Rather, our study documents the acute effects of H_2_S among a relatively young, highly educated sample of healthy adults. Thus, our results probably underestimate symptoms reported by the general community dwelling populations that include a broader age range and other health conditions.

## Figures and Tables

**Table 1:** Symptoms, environmental quality, and odor ratings.

Type of symptom	Symptom
Physical	Headache Fatigue Lightheaded Drowsy Nausea
Cognitive	Difficulty concentrating Disoriented/confused Dizzy
Eye irritation	Eye irritation (burning, dryness, or itching) Runny/watery eyes
Anxiety	Feel jittery in body Feel nervous Heart palpitations Feel tense Worried
Upper respiratory	Sneeze Nasal congestion Choking Throat irritation (burning or dryness) Nose irritation, dryness, or itching
Lower respiratory	Short of breath Wheezy Chest tightening Chest pain Coughing
Somatic control	Skin irritation or dryness Stomach ache Numbness Ear ringing Leg cramps Back pain Sweating Body aches
Environmental quality	Light intensity Ventilation Air movement Air quality Noise level Room temperature Humidity Odor level
Odor ratings	Level of irritation from odor (0–no sensation to 100–strongest imaginable); Intensity of odor (0–no sensation to 100–strongest imaginable); Pleasantness of odor (0–very pleasant to 9–very unpleasant).

Symptoms rating scale: 0, barely detectable/no sensation; 100, strongest imaginable.

**Table 2:** Postural sway length for H_2_S exposure concentration groups [ppm (mean ± SD)].

	Sway length (cm)					
	0.05 ppm		0.5 ppm		5 ppm	
Test condition	Preexposure	Exposure	Preexposure	Exposure	Preexposure	Exposure
Eyes open	30.98 ± 6.76	30.81 ± 6.67	31.20 ± 7.64	32.06 ± 11.17	30.40 ± 6.20	30.63 ± 7.03
Eyes closed^a^	41.15 ± 12.20	40.08 ± 13.15	42.43 ± 12.21	42.45 ± 16.78	40.80 ± 12.13	39.85 ± 11.29
Eyes open, soft surface^b^	39.67 ± 8.81	37.14 ± 8.04	38.96 ± 7.96	36.00 ± 7.35	39.56 ± 10.13	36.80 ± 8.06
Eyes closed, soft surface^c^	64.56 ± 22.03	61.24 ± 21.42	68.33 ± 26.56	62.76 ± 22.13	67.44 ± 24.84	62.23 ± 22.35
Right leg^d^,^e^,^f^	85.72 ± 24.50	85.41 ± 24.21	90.98 ± 26.99	83.14 ± 23.84	89.32 ± 22.88	84.45 ± 23.77
Left leg^g^	88.19 ± 26.38	78.42 ± 22.39	86.15 ± 24.96	83.05 ± 21.19	87.19 ± 21.85	79.38 ± 20.70

Significance tests for pairwise comparison of exposure conditions control for baseline performance in each exposure condition before performing the comparisons.

a Exposure main effect (*F* = 2.56; df = 2, 141; *p* = 0.08).

b Time effect: preexposure > exposure (*F* = 25.30; df = 1, 212; *p* < .0001).

c Time effect: preexposure > exposure (*F* = 18.83; df = 1, 212; *p* < 0.0001).

d Exposure × time interaction: (*F* = 4.58; df = 2, 211; *p* = 0.01).

e 0.5 ppm < 0.05 ppm (*F* = 9.03; df = 1, 211; *p* = 0.01).

f 5 ppm < 0.05 ppm (*F* = 3.26; df = 1, 211; *p* = 0.073).

g Exposure × time interaction (*F* = 2.34; df = 2, 212; *p* = 0.10).

**Table 3:** Postural sway area for H_2_S exposure concentration groups [ppm (mean ± SD)].

	Sway area (cm^2^)					
	0.05 ppm		0.5 ppm		5 ppm	
Test condition	Preexposure	Exposure	Preexposure	Exposure	Preexposure	Exposure
Eyes open^a^	1.72 ± 0.81	1.85 ± 1.15	1.78 ± 0.80	1.91 ± 1.22	1.84 ± 0.97	1.68 ± 0.94
Eyes closed^b^	2.32 ± 1.46	2.36 ± 1.91	2.65 ± 1.60	2.80 ± 2.64	2.64 ± 1.67	2.40 ± 1.61
Eyes open, soft surface^c^	2.48 ± 1.20	2.11 ± 0.75	2.59 ± 1.32	2.20 ± 0.97	2.81 ± 1.43	2.32 ± 0.98
Eyes closed, soft surface	5.23 ± 2.51	4.86 ± 2.75	5.63 ± 2.98	5.03 ± 2.82	5.71 ± 3.77	5.19 ± 3.22
Right leg	3.99 ± 1.69	4.11 ± 1.64	4.34 ± 1.70	3.85 ± 1.59	4.27 ± 1.66	4.19 ± 2.08
Left leg	4.40 ± 1.85	3.94 ± 1.65	4.40 ± 1.78	3.96 ± 1.61	4.26 ± 1.44	3.85 ± 1.92

a Exposure × time interaction (*F* = 2.52; df = 2, 212; *p* = 0.08).

b Exposure main effect: 0.05 ppm > 0.5 ppm; 0.05 ppm > 5 ppm (*F* = 5.28; df = 2, 141; *p* = 0.006).

c Exposure main effect: 0.05 ppm > 0.5 ppm; 0.05 ppm > 5 ppm (*F* = 4.49; df = 2,141; *p* = 0.01)
